# Integrating genetics and transcriptomics to study major depressive disorder: a conceptual framework, bioinformatic approaches, and recent findings

**DOI:** 10.1038/s41398-023-02412-7

**Published:** 2023-04-19

**Authors:** Emily M. Hicks, Carina Seah, Alanna Cote, Shelby Marchese, Kristen J. Brennand, Eric J. Nestler, Matthew J. Girgenti, Laura M. Huckins

**Affiliations:** 1 Pamela Sklar Division of Psychiatric Genomics, Departments of Psychiatry and of Genetics and Genomic Sciences, Icahn School of Medicine at Mount Sinai, New York, New York 10029 USA; 2 Nash Family Department of Neuroscience, Friedman Brain Institute, Icahn School of Medicine at Mount Sinai, New York, New York 10029 USA; 3 Department of Genetics, Yale University School of Medicine, New Haven, CT 06511 USA; 4 Department of Psychiatry, Yale University School of Medicine, New Haven, CT 06511 USA

**Keywords:** Personalized medicine, Depression

## Abstract

Major depressive disorder (MDD) is a complex and heterogeneous psychiatric syndrome with genetic and environmental influences. In addition to neuroanatomical and circuit-level disturbances, dysregulation of the brain transcriptome is a key phenotypic signature of MDD. Postmortem brain gene expression data are uniquely valuable resources for identifying this signature and key genomic drivers in human depression; however, the scarcity of brain tissue limits our capacity to observe the dynamic transcriptional landscape of MDD. It is therefore crucial to explore and integrate depression and stress transcriptomic data from numerous, complementary perspectives to construct a richer understanding of the pathophysiology of depression. In this review, we discuss multiple approaches for exploring the brain transcriptome reflecting dynamic stages of MDD: predisposition, onset, and illness. We next highlight bioinformatic approaches for hypothesis-free, genome-wide analyses of genomic and transcriptomic data and their integration. Last, we summarize the findings of recent genetic and transcriptomic studies within this conceptual framework.

## Introduction

MDD is a debilitating disorder and the second leading contributor to chronic disease burden globally [1, 2]. From large genomics studies and postmortem brain transcriptomics studies, MDD is thought to emerge from a complex interplay of genetic and environmental factors [3–5] and can be characterized by dysregulation of the brain transcriptome [6, 7]. Compared to diseases of peripheral tissues, the study of depression is complicated by our limited access to pathological brain tissue. Thus, functional genomic studies, animal models of chronic stress, and induced pluripotent stem cells present complementary avenues for exploring the transcriptional signatures of depression at different points of pathogenesis. There is surprisingly little cross-talk between these fields [8], perhaps due to challenges in interpreting results in relation to each other. Integration of findings from these different perspectives will produce a more comprehensive understanding of the transcriptional dynamics of depression and, within a greater multiscale framework, facilitate the discovery of effective therapies.

Depression research is further challenged by an inadequate diagnostic definition, which captures a spectrum of different symptom profiles [9, 10], longitudinal courses [11, 12], and comorbidities [13] that likely represent several distinct biological etiologies [14] (reviewed in [15–19]). Extensive discussion of MDD heterogeneity and subtyping efforts is beyond the scope of this review; however, these key concepts represent a crucial orthogonal perspective complementary to the framework presented here.

In this review, we discuss a conceptual framework that outlines approaches for exploring the transcriptomic dynamics of MDD from predisposition to onset to illness. We next highlight bioinformatic approaches for the interpretation of high-dimensional genomic and transcriptomic data and their integration. Last, we summarize recent findings of MDD transcriptomics using this conceptual framework.

## Transcriptional dynamics of depression

**Fig. 1:**
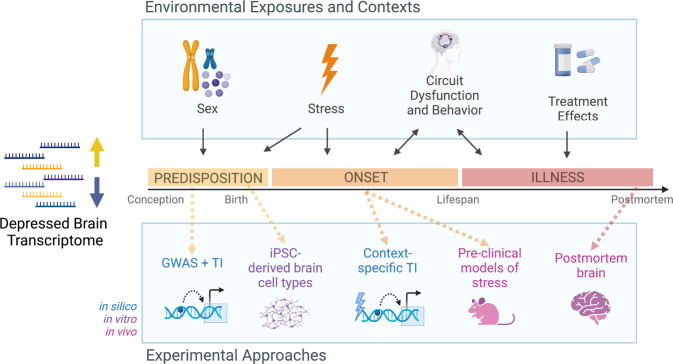
Conceptual framework for exploring transcriptional dynamics of MDD in the brain. Transcription dysregulation associated with depression is dynamic from predisposition to onset to illness; it is influenced by environmental exposures, contexts, and genetics, and can be studied from a variety of experimental approaches. GWAS genome-wide association study, TI transcriptomic imputation, iPSC induced pluripotent stem cells. Created with BioRender.com.

From a molecular perspective, depression is a multi-gene syndrome; disease pathology arises from very large numbers of small changes compounding across the genome, affecting the expression of hundreds of genes, and these processes are influenced heavily by a lifetime of adverse experiences [3, 4, 6, 7, 20–22]. As such, the onset of depression at the transcriptomic level is thought to be the result of an accumulation of genetic risk and molecular responses to environmental exposures that converge on specific functional gene networks leading to persistent transcriptome-wide dysregulation. We propose a conceptual framework that describes brain gene expression dynamics throughout the progression of MDD from predisposition to onset to illness to remission to relapse due to dynamic, fluctuating contributions of genetic risk, stress exposure, neural circuit dysfunction [23], and treatment [24] and is further modulated by age, sex, tissue type, brain region, and cell type, among other contexts (Fig. 1). Through the study and integration of the full range of transcriptional dynamics of MDD, we can develop a clearer picture of the molecular pathophysiology of MDD and efficiently identify therapeutic targets.

### Predisposition

Individual variation in transcription regulated by genomic variants may increase liability for developing MDD. Though depression is only moderately (~35%) heritable [25], over 150 common single nucleotide polymorphisms (SNPs) have been identified in association with MDD in large genome-wide association studies (GWAS), including more than 1 million individuals (*n* = 1,154,267; 340,591 cases). Historically, GWAS findings have also been limited by a lack of interpretability from SNP to function; however, functional genomics methods have propelled the mapping of genetic risk variants to systems and multi-omic outcomes (reviewed in [26]). SNPs associated with biological phenotypes, called quantitative trait loci (QTLs), make up the backbone of these analytic genomics methods. Cis-expression QTLs (SNPs associated with expression of a nearby gene; eQTL) are used to statistically map putative functional outcomes of carrying depression risk alleles [27]. It is worth noting that recent studies have detected increasing genetic mosaicism in postmitotic neurons [28], which may complicate the interpretation of GWAS findings normally collected from peripheral tissues.

Transcriptomic imputation (TI) methods jointly model eQTLs to impute genetically regulated gene expression (GREx) from genotype information [29]. In other words, these models impute an unperturbed and tissue-specific baseline transcriptional signature on an individual-level or using GWAS summary data to identify genes associated with genetic risk variants. Compared to postmortem brain samples, genetic studies have the advantage of increased statistical power due to very large sample sizes, as well as a base level of causal directionality since the genome is largely static through the lifespan.

The effect of genetic predisposition for MDD on cellular phenotypes and their development may be captured using human-induced pluripotent stem cell (hiPSC) methods, which conserve donor genetic backgrounds across a wide variety of MDD-relevant cell types, including hiPSC-derived serotonergic [30], glutamatergic [31], and GABAergic [32] neurons, microglia [33], and astrocytes [34]. Notably, reprogramming to the hiPSC state removes epigenetic markers of age [35] and is therefore presumed to likewise erase epigenetic signatures associated with environmental exposure and disorder pathology (e.g., stress, disease progression, medication use, etc. [36]). Moreover, hiPSC-derived neurons are widely accepted to most resemble their fetal counterparts [37–42]. Consequently, transcriptional differences observed in hiPSC-derived cells characterize the consequence of donor genetic risk alone and cannot account for those risk factors for depression that are not heritable. It is hypothesized that the conservation of epigenetic markers may be achieved through direct reprogramming strategies [43] if donor-specific epigenetic profiles, such as with aging [44, 45], are of interest. While experimentally powerful, hiPSC models cannot yet reliably model the cell–cell and circuit-level interactions of an intact brain, though innovations in organoid tissue culture techniques are closing this gap [46, 47].

CRISPR engineering of isogenic hiPSC lines will be useful for the direct perturbation of SNPs and genes implicated in MDD. For noncoding variants with known eQTL associations, CRISPR-activation [48] and CRISPR-repression [49] technology may be used to perturb genes in their direction of effect [50]. Multiplexing of these strategies allows for the parallel engineering of multiple perturbations simultaneously [51]. Such strategies have successfully been used to resolve the effects of common variants in schizophrenia [52] and are likely to resolve cell type-specific and context-dependent small effect sizes of individual genetic predispositions in depression.

### Onset

A high genetic risk for depression does not guarantee the onset of the disorder. Similarly, while psychosocial stressors increase the risk of developing depression, stress alone is not pathogenic [53, 54]. Indeed, many (perhaps even most) people experience stressful life events without developing psychopathology. Depression, along with other stress-related disorders, is thought to manifest when coping strategies following stress exposure are insufficient or dysregulated [8]. At the molecular level, MDD onset occurs through complex interactions of genes and environment, which can be studied using cross-sectional human genetic data [55–57], human stem cell-based models [58–60], and animal models of stress [61–63].

Transcriptomic responses to stress can be genetically regulated [64] and can be studied with large-scale genomics data. eQTLs vary by environmental context, and have been differentially detected [65, 66]across tissues, cell type- [67], and perturbation-specific [68–70] contexts (reviewed in [26]). Joint genetic and environmental regulation of expression (GxE-REx) may be inferred from the context in which eQTLs are detected (e.g., stress), thus capturing GxE regulatory mechanisms underlying that context. These QTLs can be used to create TI models [29] to predict context-specific transcriptomic responses from genotypic data. Such approaches have been previously applied to detect tissue-specific mediators of schizophrenia [71, 72], PTSD [73], bipolar disorder [74], and anorexia nervosa [75], and can be used to detect transcriptomic profiles that confer susceptibility to MDD with exposure to stress.

Cellular mechanisms of stress response can be assessed across diverse cell types and donor-specific genetic backgrounds in vitro using hiPSC methods [58]. Organoid-based approaches add an additional layer of complexity by modeling cell–cell interactions that better replicate the cellular environment in vivo [58]. When combined with CRISPR engineering to facilitate isogenic comparisons [76], phenotypic assessment of subtle gene–environment interactions between variants/genes and pertinent stressors, across cell types of interest, are testable at scale [51]. For instance, recent advances in pooled-hiPSC screening techniques such as census-seq are capable of population-scale screening of ~100 donors in a single dish [77]. As noted above, hiPSC-derived neurons are developmentally immature and most resemble their fetal counterparts [37–42], and so particularly useful platforms for screening fetal impacts of maternal exposures or early childhood exposures [78] (e.g., maternal immune activation, reviewed in [79]; adverse childhood events [80]).

It is debated whether and to what degree animal models recapitulate depression, and many criteria have been proposed to assess their validity, such as face, construct, and pharmacological validity (reviewed in [81]). However, animal models need not (and cannot) recapitulate the entire human condition, but instead are most useful in establishing the underlying biology of relevant aspects of a disorder to the degree that is impossible in humans [82]. Thus, in depression research, rodent models are best fit for characterizing brain transcriptional responses to stress and investigating mechanisms of gene–environment interactions to identify mechanistically informed therapeutic targets. Interestingly, chronic variable stress, social isolation, and chronic social defeat stress paradigms each capture distinct aspects of postmortem transcriptional signatures from the prefrontal cortex and nucleus accumbens of depressed patients [21], which establishes the utility of such models to study molecular features of depression. Another crucial contribution of animal models, which is very difficult to discern from human brain transcriptomic data alone, is whether a given molecular change or set of changes are pathological (i.e., they contribute to behavioral abnormalities) or instead are adaptive (i.e., they contribute to homeostatic responses to overcome the stress, that is, they underlie resilience). Several rodent chronic stress models can differentiate genes associated with stress susceptibility versus stress resilience and thereby aid in the functional interpretations of transcriptomic data [83]. Details on current approaches to model depression endophenotypes and assess depressive behaviors are reviewed elsewhere [84].

### Illness

Lastly, and most clinically relevant, are human tissues, which can be used to study the stable transcriptional changes that persist in depression. Postmortem brain tissues have been used to study the transcriptomes of depressed patients and illustrate the transcriptomic dysregulation in depression generally using case-control status. However, a few notable postmortem brain studies include deeper patient phenotyping than case-control status and have identified molecular associations with additional dimensions, such as exposure to antidepressant exposure [85, 86] and childhood trauma [87]. Further, postmortem samples represent ‘end stage’ disease that may not recapitulate gene expression patterns during illness; moreover, postmortem gene expression may differ substantially from living.

One major goal of transcriptomic studies, aside from biomarker discovery, is to identify causal, pathological mechanisms that lead to disorder; however, postmortem analyses are limited in their ability to identify causal directionality. Though observed dysregulated genes may unidirectionally contribute to the circuit dysregulation and symptoms associated with MDD, it is likely that other transcriptomic relationships are bidirectional: transcriptomic dysregulation influences neuronal physiology and circuit function, and in the opposite direction, circuit dysfunction and altered neuronal activity influence transcriptional dysregulation via activity-dependent molecular mechanisms (reviewed in [88]). Despite their limitations, postmortem studies are important for assessing the long-term transcriptional effects of depression and antidepressants, and animal chronic stress models can be used to validate these findings [21]. As sample sizes increase for this important population, we will have greater power to identify patterns within the heterogeneity of depression.

Peripheral tissues like blood plasma are a noninvasive, surrogate source of transcriptomic data in living MDD patients, which can be used to identify clinical biomarkers. There is the expectation that blood gene expression is mostly not reflective of brain gene expression, with cross-tissue correlation in gene expression levels ranging from 0.25 to 0.64 [89]. Thus, blood transcriptomes are less likely to provide etiological insight into depression biology so long as the brain is considered the primary source of pathology. However, mechanisms of brain–immune interactions suggest that peripheral immune gene expression bidirectionally affects brain transcription and may contribute to neuropsychopathology [83, 90, 91]. As researchers identify transcriptional dynamics between blood and brain, blood transcriptional profiles remain most useful as a surrogate for biomarkers of depression and treatment response [92].

## Bioinformatic approaches and data integration

**Fig. 2:**
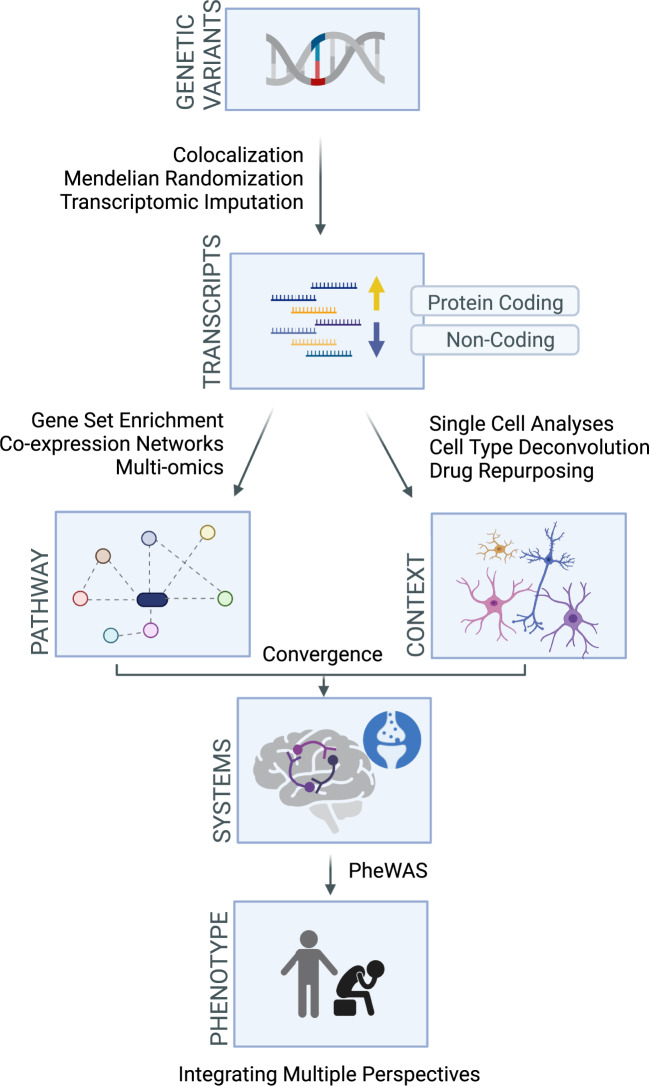
Multiscale bioinformatic approaches to study depression genomics and transcriptomics. Created with BioRender.com.

As sequencing technologies have become more affordable, large hypothesis-free transcriptomic studies have gained popularity and allowed for the investigation of large-scale transcriptional patterns. With these large datasets come increasingly complex tools to derive biological meaning and integrate these data to identify key patterns of dysregulation and, ultimately, prioritize novel therapeutic targets. In this section, we will highlight methods for analyzing these large genetic and transcriptomic datasets and discuss approaches for integrating findings from these various perspectives (Fig. 2). Table 1 summarizes the use of these analytical methods in the context of our conceptual framework and provides examples of studies for which these methods are employed.

**Table 1:** Summary of analytical methods in the context of the conceptual framework (predisposition, onset, illness) with examples of data sources and studies for which these methods are employed.

Perspective	Data source	Analytical method	Examples
Predisposition	Human genotype	GWAS	[3, 4]
	Human GWAS summary data and postmortem-brain-derived QTLs	Colocalization	[158]
		Mendelian randomization	[3]
		Transcriptomic imputation	[20, 158, 164, 218]
		Drug repurposing analyses	[3, 20, 162]
	hiPSC-derived brain cell transcriptomic data	Gene set enrichment	[59, 60, 169]
Onset	Mouse genotype and brain transcriptomic data	Context-specific QTL analysis	[178]
	Human genotype and transcriptomic data	Context-specific QTL analysis	[64, 179, 180]
	hiPSC-derived cells + glucocorticoids	Context-specific QTL analysis	[58]
		Gene set enrichment	[58, 181, 182]
	Mouse brain transcriptomic data + stress	Gene set enrichment	[61, 62, 183, 184]
		Co-expression networks	[61, 183]
Illness	Human postmortem brain single cell/nuclei RNA-seq	Gene set enrichment	[207]
		Co-expression networks	[207]
		Cell type deconvolution	[207]
	Human postmortem brain transcriptome	Gene set enrichment	[6, 7, 208]
		Co-expression networks	[6, 7]
		Cell type deconvolution	[208]

### Variant to gene

While GWAS has uncovered hundreds of common variants associated with depression, we have yet to understand how most of these variants contribute to disorder risk. Functional genomics methods, such as Mendelian randomization, colocalization, and TI, aim to bridge this functional gap by mapping SNPs to gene-level associations using reference eQTL data. Young et al. review the breadth of functional genomics tools for mapping genetic variants to gene-level and beyond to systems-level outcomes [26].

Briefly, SNP-mapping algorithms can use a proximity or QTL-based approach to translate SNPs to genes. Proximity-based approaches (e.g., MAGMA) map SNPs to genes based on proximity to a gene body. QTL-based mapping approaches are a more dependable tool for mapping because they use existing associations between genotype and gene expression from a reference dataset (e.g., GTEx [65] or psychENCODE [93]). Colocalization analysis asks whether a phenotype-associated risk SNP and an eQTL for a gene originate from the same genomic locus. As a complementary approach, Mendelian randomization (MR) uses the SNP associations as instrumental variables in a mock randomized control trial to infer causal directionality between gene expression and a trait. While neither of these methods can definitively parse pleiotropy from causal associations, genes at the intersection of both methods make high-confidence targets candidates for functional validation [94].

While these methods are useful for mapping single SNP associations, a multi-SNP model for gene expression more closely captures regulatory biology. Predictor models of gene expression are built to enable the imputation of baseline genetically regulated transcription (GREx) from genotype information (TI; e.g., PrediXcan [29]). Though slightly different in implementation, several TI models have been built to impute gene-level associations with a trait using only GWAS summary statistics (i.e., S-PrediXcan, TWAS [95, 96]), which drastically improves biological interpretability of GWAS associations in a tissue- and, soon, cell type-specific manner.

### Gene to pathways

Genes do not act in isolation to predispose, produce or perpetuate complex disorders like MDD. An important step in understanding the neurobiology of MDD is to study how individual ‘risk’ genes identified from GWAS or differentially expressed genes identified from postmortem brain data may converge on known biological pathways. Approaches to interpret gene-level associations at the pathway level include functional gene set enrichment and co-expression networks. From a statistical standpoint, these pathway-level analyses reduce the dimensionality of large gene-level analyses by grouping dependent gene expression into functional gene sets or co-expressed modules. Identification of pathways involved in depression neurobiology allows for the translation of long lists of genes into biologically interpretable results.

#### Gene set enrichment

Gene set enrichment analyses consider the membership of individual genes in known functional pathways. Using gene sets references such as GO [97] and KEGG [98], hundreds of tools have been developed to test the enrichment of target genes in known functional pathways or structures with overrepresentation analyses or threshold-free gene class scoring methods [99]. Most of these methods consider each gene in a gene set with equal weight. Pathway topology-based methods, however, attempt to quantify the importance of each gene to a pathway to improve the accuracy of enrichment analyses [100]. Current gene set methods are also limited by a lack of specificity; significant differential expression of a single gene can lead to a reported enrichment of all overlapping and/or nested gene sets that contain the gene. Several groups attempt to address this challenge using a gene set network approach [101] or by down-weighting genes with high levels of overlap [102]. Despite limitations, gene set enrichment analysis is a powerful and accessible tool for interpreting large gene lists and identifying patterns of pathway-level dysregulation.

#### Co-expression networks

Another approach to transition from a gene- to systems-level understanding of MDD transcriptomic associations is to interpret transcript-level findings in the context of co-expression networks. A co-expression network is a representation of gene expression similarity across samples, visualized by nodes (genes) and edges (molecular interactions or statistical associations) [103]. Co-expressed transcripts in a network are thought to represent biological relationships, known as the ‘guilt by association’ principle [104], and are often enriched for cell type-specific markers that can be used to annotate networks’ particular cell types. In addition to biological co-regulation, co-expression relationships can be the result of other biological or technical influences, which require careful quality control [105–107]. Networks also show limited reproducibility across network detection methods [108–110], suggesting either (1) different co-expression network methods capture complementary gene relationships of differing resolution, or (2) network results are sensitive to transcriptional noise [111]. A final consideration is that in the absence of a gold standard “known” human biological co-expression network, it is difficult to determine the effectiveness of a novel network approach. Where possible, it is encouraged that gene–gene relationships be additionally validated through comparison to independent datasets of molecular interactions or functional validation. Despite the limitations, co-expression network analysis is a powerful tool to organize individual gene results at the level of interpretable biological pathways and identify putative drivers of transcriptional organization patterns.

#### Epigenomics/multi-omics

Because of its role in integrating environmental and genetic factors, epigenetic regulation has been investigated as a key molecular player in the pathogenesis of depression [112, 113]. Studies integrating epigenetic changes such as histone modifications and chromatin landscape in other brain disorders have revealed mechanisms of dysregulation at multiple interconnected -omic levels [114–116] (reviewed in [117]). Incorporating other -omics data, such as proteomics and epigenomics, and -omics on a broader scale, such as gut microbiome, neuroimaging data, and data on lifetime environmental exposures (i.e., the exposome) [118], when brought together, can illustrate a more comprehensive view of disorder mechanisms paralleling the complexity of depression compared to transcriptomics alone.

Studies integrating genomics and neuroimaging have been particularly fruitful for identifying genetic–brain structural associations within MDD, including dimensions of sex specificity, antidepressant and trauma exposures, and will aid in translating genomic findings to the context of brain structure and function and in disentangling MDD heterogeneity [43, 119, 120]. Specific methods for data integration across multimodal data are reviewed elsewhere [121, 122].

### Gene to context

Gene expression is specific to various contexts such as tissues, cell types, developmental stage, and sex [123], as well as environmental contexts such as exposure to stress [22] or drugs. Discerning transcriptional dynamics in various contexts allows for clearer translation of gene targets to mechanistic studies.

#### Single cell analyses

Recent advances in single cell type genomic studies have made it possible to identify the specific cells that give rise to transcriptomic changes in a particular disorder [124, 125]. This is perhaps most relevant in studying neuropsychiatric disorders as the brain contains myriad cell types with individual, specific, and spatial functions. Analysis constitutes clustering single-cell transcriptomic profiles and annotating their identities based on the expression of canonical cell type markers. Within these cell type clusters, differential expression analysis can be conducted by cell identifying both differentially expressed transcripts between cases and controls within a cell type-specific context. Additionally, pseudotime trajectories can be mapped to characterize transcriptional transitions (e.g., microglial activation) [126] across age.

One caveat to single cell type studies is a lack of transcript coverage: typically employed nuclei isolation techniques only capture 7000–9000 transcripts per cell compared to bulk tissue RNA-seq technologies that capture more than double that number. Technical difficulties isolating whole cells from postmortem tissue have made single-nuclei isolation necessary for these studies and it is becoming increasingly popular to use nuclei in parallel animal studies as well [127, 128]. While there is a high degree of concordance between nuclear and cytoplasmic RNA complement [129, 130], it is unavoidable that we are missing critical information particularly from the processes of cells where the local translation is likely disrupted [131]. On the other hand, the nuclear transcriptome better captures classes of noncoding RNAs that serve important regulatory functions [63, 132, 133]. The best practice for future studies will be a combination of bulk tissue coupled with single-cell RNA sequencing, which would allow for the identification of missing or lowly expressed transcripts.

#### Cell type deconvolution

Recently developed algorithms aim to infer cell type proportions [134] and even cell type-specific expression [135] from bulk tissue gene expression data using reference single-cell datasets (reviewed in [136, 137]). Estimating cell type proportions from gene expression data allows for testing associations of cell type proportions affected by a given trait and possibly correcting for cell type proportion alterations that may confound gene expression analyses. Inference of cell type-specific gene expression [135, 138] enables analysis of bulk tissue expression datasets with some cell type specificity, although not as reliably as directly measured single-cell data, and the reanalysis of existing bulk tissue expression data in a novel context. Deconvolved bulk datasets also provide validation of single-cell data to bridge the trade-offs in the limitations of each approach.

#### Drug repurposing

Strategies to identify new applications of existing drugs are being modeled in silico using structural and transcriptional signatures of drugs and target disorder transcriptional signatures (reviewed in [139]). These tools aim to accelerate the identification of novel pharmaceutical interventions for depression and other psychiatric disorders which lack high-confidence, etiologically informed therapies, and to potentially do so with patient-specific precision. Drug repurposing methods have been applied to transcriptomic data from a variety of neurologic and psychiatric disorders [140–143], and to genetically regulate expression profiles of MDD subtypes [20].

### Systems to phenotypes

Pathway-level and context-dependent analyses of transcriptomics highlight overall patterns of transcriptomic dysregulation in the multiple systems (i.e., synaptic, endocrine, immune) associated with depression. Tying these systems together is a continual challenge of depression research.

#### PheWAS

One approach for exploring phenotypic links to tissue-specific genomic contexts is the phenome-wide association study (PheWAS [144, 145]). Using electronic health records and genomic data from large-scale biobanks, we can begin to explore the phenotypic consequences of genetic and transcriptomic variability as predictors for hundreds of phenotypic outcome variables such as insurance billing codes (ICD codes), their translation to phenotypic groups called PheCodes [146, 147], lab results (LabWAS), or prescribed medications, for example. PheWAS of imputed expression of disorder-associated genes demonstrate pleiotropic associations and potential functional mechanisms, such as in a study of anorexia nervosa [148]. PheWAS are also useful for screening potential adverse effects to anticipate in pursuit of genes as therapeutic targets.

### Integrating transcriptomic data from multiple studies and approaches

In moving toward a comprehensive understanding of depression, many datasets and data types will need to be interpreted together. It may be useful to evaluate a degree of concordance or discordance between two sets of gene expression profiles. Rather than assigning an arbitrary cutoff and evaluating the intersection of two lists of genes, rank-rank hypergeometric overlap (RRHO) takes a threshold-free approach to visualize significance levels of overlap at all possible cutoffs with a heatmap [149]. This allows for a general overview evaluation of the degree of concordance in up- or downregulated genes between two lists. For a more rigorous evaluation of convergent signatures, there exist several meta-analytic methods to increase power for detection from similar studies, such as those based on effect size combination, *p*-value combination, and nonparametric ranking methods (reviewed in [150]). In some cases, groups of similarly expressed genes are relevant only in certain conditions but not in others. Bi-clustering algorithms take into account a second dimension of condition (or phenotype) to identify clusters of convergent gene expression observed in clusters of samples, which is useful for identifying symptom subtype-specific expression profiles, for example [151].

There are several approaches by which transcriptional signatures may be compared to genome-wide associations from genetic studies. Partitioned heritability LD score regression annotates all SNPs within a certain distance from any gene in a provided gene set (e.g., postmortem brain DEGs) and calculates whether a significant portion of SNP heritability for a trait is explained by the annotated SNPs [152]. In a complementary approach, risk SNPs can be mapped to genes and then tested for enrichment in a gene set with an overrepresentation analysis. MAGMA is a flexible tool for running such an analysis that implements competitive gene set enrichment analyses and can test for continuous gene properties such as gene expression levels [153].

**Fig. 3:**
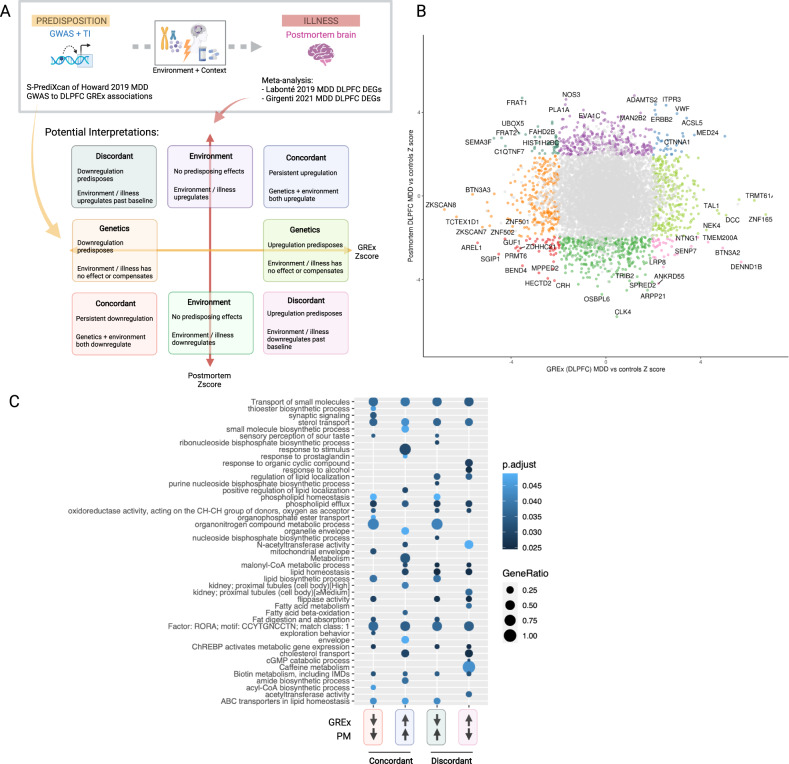
Mapping gene-level associations for depression from predisposition to illness. **A** Conceptual representation of analysis and potential interpretations for dynamic MDD gene expression associations. Predisposition: dorsolateral prefrontal cortex (DLPFC) genetically regulated expression associations were predicted with S-PrediXcan using MDD GWAS [3] and CommonMind Consortium DLPFC eQTL predictor model [71]. Illness: MDD vs healthy control differentially expressed genes [3, 4] were meta-analyzed using a fixed-effects inverse variance method. **B** *Z*-scores for GREx (predisposition) and postmortem (illness) associations of MDD vs controls are plotted. **C** Gene set enrichment (g:Profiler [155]) of top concordant and discordant genes (*p* < 0.05) from analysis in (**B**). GREx genetically regulated expression, PM postmortem. Created with BioRender.com.

We constructed a two-dimensional space within which to compare genes associated with MDD through observed vs. predicted expression studies. By definition, genes associated with MDD in predicted gene expression analyses (e.g., TWAS, PrediXcan) are identified due to germline differences in allele frequency; as such, any differences necessarily pre-date disease onset and are related to predisposition, rather than experience. Observed gene expression differences, by contrast, may reflect both predisposition to, and impact of, MDD. We directly compared association statistics from these two types of studies (Fig. 3A) to infer potential mechanisms of dysregulation.

Specifically, DLPFC GREx associations were imputed from MDD GWAS [3] with S-PrediXcan [95] using CommonMind DLPFC eQTL predictor model [71] to represent *predisposition*; and postmortem DLPFC gene expression associations were obtained by inverse variance weighted meta-analysis [154] of differential expression summary statistics in DLPFC of MDD cases versus nonpsychiatric controls [6, 7] to represent *illness*. Gene-level summary *Z*-scores are then plotted on each axis (Fig. 3B). A handful of transcripts are significantly regulated in both GREx and postmortem associations based on a loose significance threshold (*p* < 0.05), capturing broad patterns of regulation. Gene set enrichment [155] of these top concordant and discordant genes reveal patterns of dysregulation from predisposition to onset, implicating small molecule signaling, synaptic signaling, and lipid metabolism and biology, among other gene ontology terms (Fig. 3C). Interestingly, some larger gene sets show enrichment in both concordant and discordant groups which may suggest nonspecific dysregulation among these pathways (transport of small molecules, RORA transcription factor binding) that lends itself to the complexity of transcriptional dynamics (e.g., compensatory mechanisms).

## Recent findings

### Predisposition

Despite a moderate heritability of MDD (twin-based *h*^2^ = 38%; SNP-based *h*^2^ = 21–30 [25]%), depression GWAS are now approaching sufficient power to discover novel associations that contribute to depression risk. This is due in part to recent inclusions of broader self-declared definitions of depression with clinically diagnosed MDD, which are highly genetically correlated (*r*_G_ = 0.86, s.e. = 0.05) [156]—though perhaps at the cost of specificity [157]. A recent meta-analysis led by the Psychiatric Genomics Consortium (PGC) included data from PGC, 23andme, UKBiobank, and FinnGen (*n* = 807,553; 246,363 cases [3]). These analyses were later meta-analyzed with additional data from the Million Veteran Program, producing the largest published GWAS of depression to date [4]. This study includes over 1.2 million individuals of European ancestry (*n* = 1,154,267; 340,591 cases) and African ancestry (*n* = 59,600; 25,843 cases) [4] and identified 178 genomic loci associated with depression.

Depression risk SNPs are enriched in conserved, intronic, and H3K4me1 enhancer regions of the genome, and nearby genes specifically expressed in the frontal cortex or anterior cingulate cortex and in neurons [3]. Gene-level mapping of depression risk SNPs converges on genes enriched in brain tissues, pyramidal neurons, neuronal processing, synaptic structure and processing, as well as sex hormone receptor signaling [3, 4, 158, 159]. Interestingly, associations with the BTN family of immune regulators have also been linked in a few studies to depression predisposition [159, 160] as well as with the shared genetic risk for depression and insomnia [161]. Drug–gene interaction studies highlight antipsychotics, estrogen-related drugs, DRD2-targeting drugs, calcium channel modulators, and serotonin receptor antagonism as potential drugs to counteract transcriptional effects of depression risk [3, 162]. Individual genes that commonly emerge among these studies include those with existing links to depression, such as *DRD2* [4, 159, 162, 163] and *FADS1* [4, 164, 165], as well as novel genes such as *NEGR1* [4, 158, 162, 166] and *RPL31P12* [160, 166] without existing links to depression. These novel associations are promising targets for functional validation as a point of therapeutic intervention.

Studies of depression predisposition also highlight the role of risk SNPs in the context of development. Predicted DLPFC risk genes are more highly expressed in prenatal stages than postnatal stages suggesting risk genes may confer their predisposing effects during development [164]. Such findings warrant investigation of the genetic predisposition for MDD on cellular phenotypes and their development using hiPSC methods which conserve donor backgrounds across a variety of MDD-relevant cell types [167]. A handful of studies have investigated MDD-specific hiPSC transcriptomes in the context of SSRI-resistant depression [59, 60], likely due to the heterogeneous nature of disorder phenotypes (reviewed in [168]). Vadodaria et al. derived neurons from SSRI responder and nonresponder patients and found a 5HT-dependent response difference between the groups [60] and transcriptomic differences in *PCDHA6* and *PCDHA8* as mediators of this effect [59]. In another study, patient-derived cortical neurons were screened for bupropion response, finding differences in synaptic morphology and transcription between responders and nonresponders [169]. Together these studies begin to describe the transcriptomic and neural consequences of genetic risk and in utero stress exposures for developing depression.

### Onset

Large-scale gene–environment (GxE) interaction studies are necessary to wholly capture MDD genetic etiology; SNP-based heritability of MDD with reported trauma exposure (24%) is greater than the heritability of MDD without reported trauma exposure (12%). Interaction studies of childhood trauma [170], stressful life events (SLEs [171]), systematic discrimination [172], and personal traumas (e.g., sexual assault) [173–176] with MDD genetic risk have identified specific SNP interactions pointing to potential mechanisms of depression onset. Individuals with a genetic predisposition to MDD who experience these traumatic events have worsened MDD symptoms—up to twice as high as individuals without trauma exposure [170]. Using Mount Sinai’s Bio*Me* biobank, the Huckins group tested the interaction between MDD polygenic risk score (PRS) (derived from MDD PGC GWAS [3]) and 12 traumatic and stressful life events associated with MDD (TSLEs) and discovered a significant interaction effect between MDD-PRS and motor vehicle accidents (B = 159.12, *p* = 4.20 × 10^−4^ [177]). These interactions highlight that genomic and environmental factors cooperatively contribute to depression and motivate investigations of GxE interactions at the molecular level.

One approach is to identify eQTLs in the context of stress. In the periphery, Arloth et al. identified glucocorticoid receptor (GR)-responsive eQTLs enriched in GR binding sites and known depression risk variants [64]. However, the challenge remains to elucidate stress context-specific GREx in neural tissue. Emerging works address this challenge by modeling eQTL effects experimentally using massively parallel reporter assay (MPRA) approaches, and are uncovering glucocorticoid-, retinoid- and sex-specific eQTLs in neural cells in vitro and in mouse models [58, 178–180]. Such analyses integrating genetic variation hold promise for identifying mechanisms explaining individual differences in the onset of psychiatric disorders following stress. Furthermore, regulatory logic from these studies may be used to derive context-specific transcriptomic imputation models as a novel means to address GxE interactions in silico to apply to large genomic datasets.

Though the transcriptional effects of stress exposure in the brain cannot yet be measured directly in living human brains, they have been characterized in vitro using hiPSC-derived cells and in vivo in mice exposed to chronic stress. Physiological stress exposure, as mediated through the hypothalamic-pituitary-adrenal axis, can be modeled with glucocorticoids. In hiPSC-derived astrocytes, chronic glucocorticoid exposure is associated with an MDD-specific transcriptional profile enriching in GPCR signaling and synaptic processes [181]. Transcriptional signatures of glucocorticoid activation in hiPSC-derived brain organoids demonstrate lasting cell type-specific effects on neural development, specifically promoting proliferation and decreasing differentiation via dysregulation of neuronal specifying genes (e.g., *TCF4*, *AUTS2*, *PAX6*, *RELN*, *OTX2* [58]). Stress-related exposures of hiPSC-derived neurons have also been explored under different disorder paradigms, such as in PTSD, where acute glucocorticoid exposure elicits a PTSD diagnosis-dependent transcriptomic response [182]. Together, these findings demonstrate the promise of hiPSC-derived cell types combined with environmental perturbations to investigate the gene–environment interactions underlying MDD.

In mice, Bagot et al. observed key circuit-wide differences in transcriptional patterns between resilient and susceptible male mice following chronic social defeat stress [183]. Several networks of gene co-expression enriched in neuronal and synaptic transmission genes emerged in susceptible animals that are not observed in control or resilient animals across several limbic brain regions [183]. Conversely, a follow-up study identified gene networks associated specifically with resilient animals [61]. Together, these results suggest that dysregulation induced by chronic stress may be mediated by the reorganization of neuronal regulatory networks controlling synaptic transmission in several brain regions. The timing of exposure to stressful events, especially during critical periods of plasticity such as in childhood, increases the risk of developing MDD and has also been studied in mouse models. Early life stress (ELS) in rodents produces latent transcriptional changes in the ventral tegmental area, PFC and NAc that are later altered with exposure to adult stress [62, 184]. Interestingly, rather than exaggerating the transcriptomic response to ELS, adult stress produces a modified transcriptional response enriched in cholinergic signaling genes that also differs from transcriptional responses to adult stress alone [62]. This suggests that transcriptomic changes from ELS may reflect a different regulatory landscape compared to adult stress-induced depression alone and may require alternative therapeutic strategies, which coincide with known associations between childhood trauma and treatment-resistant depression [185–187].

### Illness

Extensive efforts have been made over the past decade to characterize the human MDD transcriptome within discrete brain regions and cell types using postmortem tissue. Most postmortem brain studies of depression have focused on the prefrontal cortex, amygdala, and hippocampus, regions implicated in the behavioral deficits of depression with known cellular, structural, and molecular alterations [188–194]. Numerous studies have used microarrays to profile brain gene expression [195–199] (up to 2010 reviewed in [200]). The first genome-wide studies identified significant increases in *DUSP1*, which plays a major role in neural plasticity in the hippocampus [201]. Initial studies of the prefrontal cortex of MDD subjects identified significant decreases in transcripts involved in synaptic transmission that also correlated with observed decreases in synaptic number and loss of dendritic spines and dendrites in donor-matched histological sections of the PFC [194, 202]. These studies identified deficits in neurons but lacked the resolution to identify the subtypes involved in MDD pathophysiology.

Several studies have identified deficits in somatostatin (SST) transcript levels in the subgenual anterior cingulate cortex and the amygdala of MDD patients [203–205], implicating inhibitory neurons. Based on these findings, it is hypothesized that MDD deficits in SST transcript could be a result of cellular vulnerability and that levels of SST are directly involved in cellular processes (such as protein translation [206]) that affect the synaptic output of interneurons within their circuit. Alternatively, these deficits may also be compensatory to maintain excitation/inhibition balance due to altered function of the excitatory and pyramidal neurons. Interestingly, a recent single-cell transcriptomic profiling of depressed postmortem DLPFC found that a majority of their DEG signal came from excitatory neurons (10 cell type clusters) and oligodendrocyte precursor cells (nearly half of the DEGs identified), with only modest contribution from interneurons (three clusters from SST and PV cells and three from non-SST/non-PV interneurons) and non-neuronal cells [207]. These findings suggest that we are missing critical information about the cell types contributing to the molecular pathology and highlight the need for region- and cell type-specific profiling in future postmortem studies.

Recent evidence points to sex-specific genomic differences in MDD patients. A landmark paper by the Nestler group identified largely nonoverlapping signatures in the differentially expressed transcripts of males and females with MDD across six brain regions [6]. Gene co-expression analysis also revealed differences in the transcriptomic organization of males and females with depression. Remarkably, exogenous downregulation of the female-specific MDD hub gene *Dusp6* resulted in stress susceptibility in female but not male mice and mimicked many of the transcriptomic changes observed in postmortem brains of donors with MDD. Several studies have since confirmed these findings [7, 208]. Taken together, these studies highlight the importance of sexual dimorphism in functional genomic studies [208, 209].

## Future directions

Depression is a multiscale disorder influenced by genetics, environment, and complex interactions. We propose a conceptual framework for interpreting various approaches we have available to study the brain transcriptome in depression. While access to brain tissue remains scarce, other avenues for studying the human brain are emergent. For example, the Living Brain Project is an exciting new research program taking advantage of advances in neurosurgery for novel sources of living human brain tissues for molecular profiling, though tissue would be limited to disease/disorder cases. For depression specifically, the BeCOME study will produce deeply phenotyped and multilevel data for subtyping affective disorders and developing precision therapies [210].

Integrating transcriptomic data, as reviewed here, with multiscale data, will be fruitful for depression research. Circuit-aware transcriptomic profiling [183], single-cell approaches [211], and spatial transcriptomics [212] will be useful for further contextualizing gene expression alterations within relevant circuits and cell types. Genetic imputation models, like BrainXcan, linking genetic variants to systems-level outcomes such as brain structural and connectivity phenotypes [213] and incorporating context and environmental factors [214] will propel the translation of depression risk variants to biologically relevant phenotypes with increasing complexity. Finally, large multiscale and multi-omic analyses (e.g., [215, 216],) integrating clinical phenotypes, neuroimaging and molecular phenotypes as well as peripheral phenotypes (e.g., microbiome, exposome [217]) will be invaluable for identifying systems-wide mechanisms of depression etiology.
